# Carbamazepine-Loaded Porous Microspheres for Short-Term Sustained Drug Delivery

**DOI:** 10.4103/0975-1483.62206

**Published:** 2010

**Authors:** M Rajkumar, SB Bhise

**Affiliations:** *Department of Biopharmaceutics, Biopharmaceutical Research Group, Government College of Pharmacy, Karad, India*

**Keywords:** Carbamazepine, microspheres, re-precipitation, sustained release, dissolution enhancement

## Abstract

The present investigation is aimed to prepare the porous microspheres of carbamazepine using eudragit as release retardant, compritol as core forming agent, and HPMC as re-crystallization inhibitor for short-term sustained delivery of carbamazepine. The proposed microspheres were formulated using the emulsion solvent diffusion method. The obtained microspheres were characterized for its particle size distribution, thermal analysis (DSC), crystallinity (PXRD), surface morphology (SEM), and *in vitro* drug release. The prepared microspheres were found to be optimal in terms of particle size and entrapment efficacy. However, the obtained entrapment efficacy is insufficient to deliver the high dose drug such as carbamazepine. There were no compatibility issues and the drug is partially present in crystalline form in microspheres, which were confirmed by DSC and PXRD, respectively. The time to release 50% of drug from microspheres were in the range of 0.5 - 3.0 h, which could be used to prevent the formation of dihydrate and high extent of drug release. Further investigations are required to reduce the amount of polymer in microspheres that can provide maximum drug loading and acceptable dosage form.

## INTRODUCTION

Many oral controlled drug release systems have been developed to improve drug bioavailability and release the drug for prolonged period of time.[1] The main objective of such a drug delivery system is to control the release profile of the drug and also retain the drug in gastric environment. Moreover, the drug must be released from the delivery system in the absorption window, where the drug gets maximum absorption.[2] Most of the drug delivery carrier systems such as microspheres and nanoparticles have been developed to improve the bioavailability and to enhance the pharmacokinetic properties, which can lead to improve the patient compliance.[3] Microspheres are ideal carriers for delivery of the drug in both oral and parental routes of administration.[4] Porous microspheres have been developed for not only sustaining the drug release profile but have also been used to enhance the extent of drug release.[5] Short-term sustained release microparticles have been synthesized and evaluated as a drug delivery vehicle.[6]

Carbamazepine (CBZ), an anti-epileptic drug is used in the treatment of epilepsy, trigeminal neuralgia, and bipolar disorders. The conventional CBZ tablets yield peak plasma concentration varying from 4 to 32 h. Irregular and delayed absorption of CBZ is attributed to slow dissolution rate.[7] Dissolution enhancement of CBZ can result in increase in the rate and extent of absorption and hence bioavailability. Several attempts using water-soluble carriers have been made to prepare different formulation of CBZ solid dosage forms with improved dissolution properties.[8–12] Variation in drug dissolution is also due to the availability of various physical forms of CBZ. CBZ has at least four different polymorphs (I, II, III, and IV) and a dihydrate form. Dihydrate of CBZ has one-third solubility compared to its anhydrous form. The conversion of CBZ to carbamazepine dihydrate (CBD) in the gastrointestinal tract is one of the major rate-limiting steps in bioavailability of oral dosage forms.[13] The burst release of CBZ from immediate release (IR) dosage forms lead to super-saturation of the drug in GIT and facilitates formation of CBD and hence offers poor dissolution rate and bioavailability. The best way to reduce the super-saturation and re-crystallization of CBZ in stomach is formulating it as sustained release dosage forms that prevent the burst release, super-saturation, and CBD formation. CBZ loaded microspheres were synthesized and evaluated for their pharmaceutical applicability in both oral and non-oral routes of administration. However, no short-term sustained release microspheres were reported using lipid as a carrier for CBZ. The present investigation is focused on the development of lipid-based porous microspheres for short-term sustained release delivery of CBZ.

## MATERIALS AND METHODS

### Materials

Commercial CBZ anhydrate was gifted from Bajaj Healthcare Pvt. Ltd., Mumbai. HPMC E5 and Eudragit^®^ RLPO was kindly gifted from Colorcon Asia and Degussa India, respectively. Compritol 888 ATO was generously gifted from Lupin Research Park, India. All other chemicals used were of HPLC grade or analytical grade.

### Preparation of microspheres

Porous microspheres were prepared by the emulsion solvent diffusion method established by Kawashima[14] as follows: 0.2 g of CBZ, Eudragit^®^ RLPO (0.05-0.2 g), HPMC E_5_ (0.1 g), and glyceryl benhate (0.05 - 0.2 g) were dispersed in a mixture of dichloromethane (8 ml) and ethanol (2 ml) at room temperature. The resulting solution was poured into an aqueous solution of polyvinyl alcohol (1.0 w/v%, 250 ml) at 30°C. The resultant emulsion was stirred at 500 rpm employing a homogenizer for 3 h. Subsequently, the resulting microspheres were collected and dried overnight at 40°C. Composition of optimized formulations is listed in Table 1.

**Table 1:** Composition of carbamazepine porous microspheres

Batch Code	Composition (mg)			
	CBZ	EUD**	COM	HPMC
SAM 1	200	50	200	-
SAM 2	200	100	200	-
SAM 3	200	150	200	-
SAM 4	200	200	200	-
SAM 5	200	150	50	-
SAM 6	200	100	100	-
SAM 7	200	50	150	-
SAM 8	200	50	200	100
SAM 9	200	100	200	100
SAM 10	200	150	200	100
SAM 11	200	200	200	100

### Pharmaceutical characterization

#### Particle size analysis

Particle size measurement was carried out by the optical microscopy method. The prepared microspheres were subjected for particle size analysis. Microspheres were suspended in liquid paraffin and particle size was measured.

### Encapsulation efficacy

The CBZ content in the microspheres was determined by pulverizing the CBZ-loaded microspheres (10 mg) followed by immersing them in 500 ml water with agitation at room temperature for 10 min. After filtration through a 0.45 µm membrane filter (Millipore India, Bengaluru), the drug concentration was determined at 287.5 nm using the UV-visible spectrophotometric method. All samples were analyzed in triplicate, and the encapsulation efficiency (EE) was calculated according to the following equation:

$$\%\;\mathrm{Encapsulation}\;\mathrm{efficacy}\;=\;\frac{\mathrm{WA}}{\mathrm{WT}}\times 100$$

WA - Actual drug content; WT - Theoretical drug content.

### Thermal analysis

Differential scanning calorimetric (DSC) analyses of the CBZ and microspheres were carried out by using a differential scanning calorimeter equipped with computer analyzer (Shimadzu TA -60 differential scanning calorimeter, Shimadzu Corporation, Kyoto, Japan). Samples (of 3-7 mg) were heated in a nitrogen atmosphere on an aluminum pan at a heating rate of 10°C/min over the temperature range of 60-200°C.

### Crystalline state evaluation

Powder X-ray diffraction (PXRD) patterns were traced employing an X-ray diffractometer (Philips PW 1729, Analytical XRD, Holland) for the samples using Ni filtered CuK(α) radiation (intensity ratio(α_1_/α_2_): 0.500), a voltage of 40 KV, a current of 30 mA, and receiving slit of 0.2 inches. The samples were analyzed over 2θ range of 5.010-39.990° with scanning step size of 0.020° (2θ) and scan step time of 1 s.

### Scanning electron microscopy

The surface topography of the microparticles was examined using optical microcopy and scanning electron microscope (Jeol, JSM-5200, Japan, 15 KV). Samples were coated with gold film under vacuum using a sputter coater (SPI Sputter™ Coating Unit, SPI Supplies, Division of Structure Probe, Inc., PA, USA) and then investigated.

### *In vitro* dissolution studies

Dissolution studies were carried out in 900 ml of distilled water as a dissolution medium at 37°C (n = 3) with a six flasks USP type 2 dissolution apparatus (Lab India Disso 2000, Lab India Pvt. Ltd, Mumbai, India). Freshly prepared distilled water was used as a dissolution medium because dissolution profile of CBZ was found to be independent of pH.[15] To ensure sink condition, during dissolution studies, sample equivalent to 50 mg of CBZ was subjected to the test, which is less than 30% of saturation solubility of anhydrous of CBZ.[16] Samples were taken at appropriate time interval. The volume of dissolution medium was kept constant throughout the run by replacing the removed samples with an equivalent volume of fresh dissolution medium. Samples were filtered through 0.44 µ filter (Whatman^®^, Clifton, NJ, USA), suitably diluted and analyzed at 287.5 nm by using a UV-visible spectrophotometer (Pharma spec 1700, Shimadzu Corporation, Kyoto, Japan).

## RESULTS AND DISCUSSION

### Preparation of microspheres

Microspheres prepared by the emulsion solvent diffusion method were found to be spherical in nature. The solvent diffusion has created a porous morphology of microspheres that could allow entrapping the drug molecule by the solvent evaporation method. Moreover, the porous nature of the microspheres facilitated large surface area that was available for drug release. Glyceryl behenate was used as a matrix-forming agent for preparation of microspheres. Eudragit RLPO was used as core forming polymer because it offers pH independent drug release and highly permeable film forming ability,[17] which could allow the GI fluid penetration inside the microspheres. During the preformulation studies, various operation temperatures such as 25°C, 30°C, 40°C, and 50°C were tried to get optimal spherical nature and porosity. The operation temperature of 40°C was found to be optimal. Homogenization speed was considered as critical parameter in the preparation of microspheres. Homogenization speed of 500 rpm was found to be optimal for preparation of microspheres. The speed of less than 500 rpm caused instability of emulsion and led to poor spherical shape. Increasing the homogenization speed up to 1500 did not affect the microsphere size and morphology. More than 1500 rpm leads to very fine microsphere that was very difficult to separate during the process. HPMC was used as excipient in microsphere formulation, which prevents CBD formation. The concentration of HPMC had a significant role in the formation of microspheres. No microspheres were formed in the concentration of HPMC above 200 mg in the formulation. The optimal concentration of 100 mg of HPMC was selected for further optimization. The present study revealed that the emulsion diffusion method was found to be suitable for preparation of glyceryl behenate-based microspheres that will be utilized to prepare sustained release dosage form of BCS class 2 drugs.

### Pharmaceutical characterization

#### Particle size analysis

**Figure 1:**
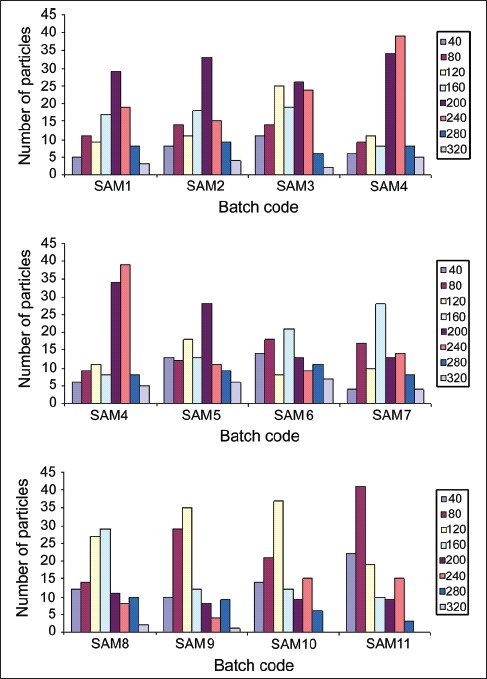
Particle size distribution of carbamazepine-loaded microspheres

The results obtained from particle size analysis revealed that arithmetic mean diameters of synthesized microspheres were in the range of 120.00-182.57 µm [Table 2]. The content of eudragit in the microspheres had influenced the particle size. High concentration of eudragit in formulation reduced the particle size of microspheres. Maximum percent frequency (26-34%) of the particles in high composition compritol (0.2 g) batches of SAM 1-5 was found to be at 200 µm. 21 and 28% frequency of particles was found with 160 µm in batch SAM_7_ and SAM_8_, respectively that pose the high concentration of eudragit to compritol ratio. The particle size analysis studies revealed that the synthesized particles were uniform in size and that can be controlled by manipulating the formulation parameters. Particle size distribution analysis indicated the non-normal distribution of microspheres in size [Figure 1].

**Table 2:** Characterization of carbamazepine loaded microspheres

Batch code	% EE + SD*	T50% (h)	A.M.D (µm)
SAM 1	31.28 + 1.93	3	182.57
SAM 2	43.40 + 0.53	2	175.35
SAM 3	58.55 + 0.88	2	164.40
SAM 4	79.10 + 1.04	1	196.33
SAM 5	59.16 + 2.12	2	167.27
SAM 6	51.38 + 1.75	3	162.37
SAM 7	47.04 + 0.32	4	170.20
SAM 8	19.14 + 1.60	2	150.79
SAM 9	23.57 + 1.52	1	131.85
SAM 10	39.35 + 1.38	0.5	142.22
SAM 11	51.13 + 0.33	0.5	120.00

* n = 3; A.M.D: Arithmetic mean diameter

### Encapsulation efficacy

Entrapment efficacy of drug-loaded microspheres was found to be in the range of 19.14-79.10% [Table 2]. Entrapment efficacy was dependent on the composition of drug as well as eudragit. Low content of eudragit and high drug composition of drug in formulation led to reduce the entrapment of drug in microsphere formulations. SAM_4_ yields maximum entrapment efficacy of 79.10%, whereas minimum entrapment efficacy of 19.04% was observed with SAM_8_. However, CBZ being a low solubility drug with high dose required low concentration of polymer and/or excipients in dosage form for better formulation development. Further research is required to improve the biopharmaceutical properties of CBZ using porous microspheres as a carrier.

### Thermal analysis

**Figure 2:**
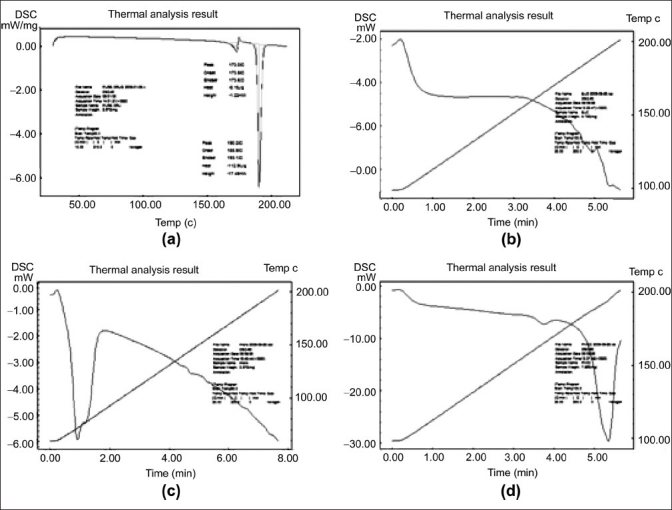
Differential scanning calorimetric of (a) Carbamazepine; (b) Eudragit^®^ RLPO; (c) Carbamazepine-loaded microsphere; (d) Physical mixture

Thermal behaviors of CBZ, polymers, physical mixtures, and microspheres were characterized by differential thermal calorimetric methods. Figure 2 shows the DSC thermogram of drug, polymers, and formulation. CBZ showed a first melting endothermic peak at 175.61°C with the fusion enthalpy of 13.7 J/g followed by a second endothermic peak at 191.70°C with the fusion enthalpy of 105.88 J/g. These two endothermic peaks correspond to form III and I of CBZ, respectively.[18] DSC thermogram of eudragit revealed that there were no endothermic or exothermic peaks, which indicated the amorphous nature and stability of the polymer in the operation temperature. Glyceryl behenate does not show any characteristic peak in the temperature range of 100-200°C. The reported melting point of glyceryl behenate is 70°C that was observed with DSC thermogram of drug-loaded microspheres carried out in the temperature range of 60-200°C. The physical mixture formulation has shown endothermic peak at 191°C that indicated the melting of CBZ. CBZ was not soluble in melted liquid glyceryl behenate and hence the drug was found to be in the suspension form above the melting point of glyceryl behenate. Further studies such as hot stage microscopy are required to confirm this thermal event of physical mixture. There was no endothermic peak at 191°C in solid dispersion formulation. The absence of endothermic peak at 191°C revealed that the drug-presented formulation was found to be of amorphous nature. Another possible reason for the absence of endothermic peak in solid dispersion formulation could be entrapment of drug in the microsphere formulation.

### Crystalline state evaluation

**Figure 3:**
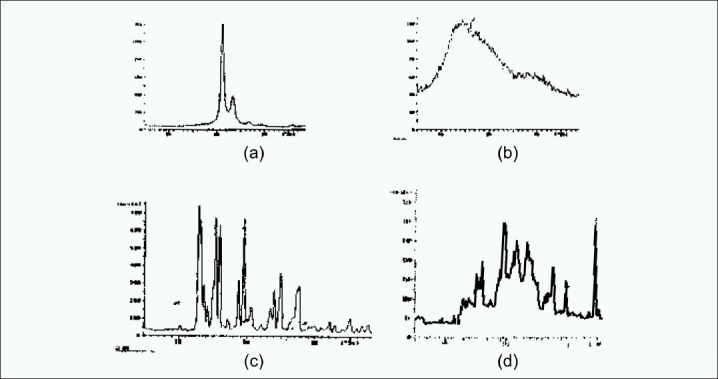
Powder X-ray diffraction of (a) Compritol; (b) Eudragit^®^ RLPO; (c) Carbamazepine; (d) Carbamazepine-loaded microsphere

The solid states of CBZ, polymers, and microspheres of CBZ were studied by XRD [Figure 3]. CBZ was detected by low-intensity diffraction peak at 2θ values of 10.105° (d value of 8.746), 13.270 (d value of 6.666) and 18.710° (d value of 4.738).[19] Eudragit does not show any characteristic peak in PXRD thermogram. The PXRD pattern of eudragit revealed the amorphous nature of the polymer. The PXRD pattern of glyceryl behenate had shown that two characteristic peak 14.470° (d value of 6.116) and 21.070° (d value of 4.213) was comparable with compritol PXRD. Moreover, PXRD data revealed crystalline nature of the polymer. The reduction in peak intensity was observed with microsphere formulation stating low crystalline nature of the drug on the surface of the micropsheres. PXRD data results confirmed that the drug present in microspheres was of either low crystalline nature or entrapped in microspheres.

### Scanning electron microscopy

**Figure 4:**
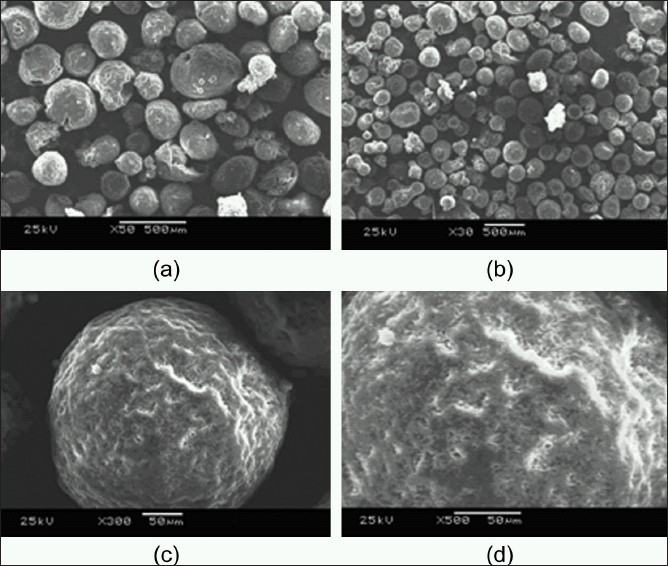
Surface morphology images of carbamazepine loaded microsphere (a) ×50; (b) ×30; (c) ×300; (d) ×500

Figure 4 illustrates the scanning electron microscopy of the microspheres. The prepared microspheres were spherical in nature and the size of the microspheres was not found to be uniform. The surface morphology analysis revealed porous nature of the microsphere that could be useful to increase the surface area and dissolution. CBZ micro-crystals appeared in the surface of the microspheres that may give the initial burst release and the drug entrapped in the microspheres provided sustained release dissolution profile. The solid state SEM analysis also revealed that the prepared microspheres were found to devoid of aggregation particles and hence poses negligible surface charges, and hence the microspheres were found to be physical stability. The high concentration of PVA, which was used in the aqueous phase during the preparation of microspheres may be one of the suggested reason for reducing the particle surface charge and hence provided aggregate free microspheres. Prepared microspheres may have excellent flow properties and hence there was no need of hydrophobic lubricants and glidants for further pharmaceutical dosage form development.

### *In vitro* dissolution studies

**Figure 5:**
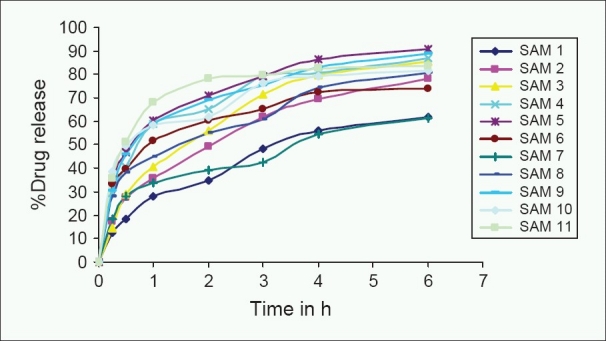
*In vitro* dissolution profiles of carbamazepine-loaded microspheres

Figure 5 illustrates the *in vitro* dissolution profile of microspheres. The solubility of CBZ was found to be pH independent and hence *in vitro* dissolution test was carried out using distilled water as the dissolution medium. CBZ-based microspheres were designed to prevent the re-crystallization of the drug by sustaining the drug release from the dosage form. Microspheres were designed to release the drug in the short-term sustained release profile, so that the drug release profile was monitored for 6 h. The distal part of GIT has less volume of the viscous fluid that may also retard the drug release profile of BCS class 2 drugs such as CBZ. Prepared microspheres had shown the drug release profile for 6 h. Prepared microspheres showed *T*_50%_ more than 1 h, which may prevent the re-crystallization of CBZ in GIT [Table 2]. The maximum release of 90.8% was observed with SAM5 formulation, which contains low glyceryl behenate and high proportion of eudragit. Glyceryl behenate being a lipophilic polymer retarded the drug release from the microspheres. High permeability of Eudragit RLPO increased the drug release in sustained manner. Minimum release of 61.9% within 6 h was achieved with high content of glyceryl behenate. The drug release profile was dependent on the concentration of both the polymers. However, high quantity of eudragit had reduced drug release from the microspheres. The mechanism for low concentration of glyceryl behenate with high content of eudragit batch (SAM 5 -SAM 7) is not known. HPMC was used as excipient in microspheres (SAM 8-SAM 11) to reduce the crystallinty and diffusion layer thickener. HPMC increased the extent of drug release up to 20% when compared SAM 1 formulation with SAM 8 formulation. However, there was no effect of HPMC on the formulation containing more than 0.75 g of eudragit. *In vitro* drug dissolution studies revealed that the prepared microspheres have optimal drug release profile and it can be used for further evaluation.

## CONCLUSION

The present investigation was aimed to prepare short-term sustained release microspheres for delivery of CBZ that will provide controlled drug release and prevent the re-crystallization of drug in GIT. The emulsion solvent diffusion method was utilized in the preparation of CBZ microspheres. The microspheres prepared by this method were found to be of non-uniform in size and provided better-controlled release profile of CBZ that could prevent the re-crystallization and CBD formation. The drug entrapment in microspheres was found to be insufficient to prepare pharmaceutically acceptable. The drug release from microspheres was found to be dependent on the concentration of eudragit as well as compritol in microspheres. Prepared microspheres were found to be porous in nature that offered high surface area for dissolution, which may offer higher extent of drug release. However, the effect of polymer on porosity was not investigated in this present work. Selected excipients were compatible with drug that was characterized by DSC studies. CBZ was found to be partly crystalline in state and/or entrapped in the microspheres system, which was confirmed by PXRD studies. CBZ being a high dose drug required large quantity of excipient that may be the hurdle for preparation of sizeable dosage form. Further investigations are required to reduce the amount of polymer in microspheres that can provide maximum drug loading and acceptable dosage form. More, *in vivo* behavior of such dosage forms should be performed to prove their clinical efficacy.
